# Prolonged Exposure to Social Stress Impairs Homeostatic Sleep Regulation

**DOI:** 10.3389/fnins.2021.633955

**Published:** 2021-02-22

**Authors:** Basma Radwan, Alvaro Yanez Touzet, Soaad Hammami, Dipesh Chaudhury

**Affiliations:** ^1^Department of Biology, New York University Abu Dhabi, Abu Dhabi, United Arab Emirates; ^2^MBChB Medicine, The University of Manchester, Manchester, United Kingdom

**Keywords:** homeostatic sleep, slow wave amplitude, NREM sleep, chronic social defeat, sleep pressure, process S

## Abstract

Stress and sleep are tightly regulated as a result of the substantial overlap in neurotransmitter signaling and regulatory pathways between the neural centers that modulate mood and sleep-wake cycle. The chronicity of the stressor and variability in coping with it are major determinants of the psychiatric outcomes and subsequent effect on sleep. The regulation of sleep is mediated by the interaction of a homeostatic and a circadian process according to the two-process model. Chronic stress induces stress-related disorders which are associated with deficient sleep homeostasis. However, little is known about how chronic stress affects sleep homeostasis and whether the differences in adaptation to stress distinctively influence sleep. Therefore, we assessed sleep homeostasis in C57BL6/J mice following exposure to 15-d of chronic social defeat stress. We implemented wake:sleep ratio as a behavioral correlate of sleep pressure. Both stress-resilient and stress-susceptible mice displayed deficient sleep homeostasis in post-stress baseline sleep. This was due to poor temporal correlation between frontal slow wave activity (SWA) power and sleep pressure in the dark/active phase. Moreover, the buildup rate of sleep pressure in the dark was lower in susceptible mice in comparison to stress-naïve mice. Additionally, 4-h SD in the dark caused a deficient sleep recovery response in susceptible mice characterized by non-rapid eye movement (NREM) sleep loss. Our findings provide evidence of deficient homeostatic sleep process (S) in baseline sleep in stress-exposed mice, while impaired sleep recovery following a mild enforced wakefulness experienced during the dark was only detected in stress-susceptible mice. This alludes to the differential homeostatic adaptation to stress between susceptible and resilient mice and its effect on sleep regulation.

## Introduction

Sleep is ubiquitous across many diverse species such as vertebrates, arthropods, nematodes and even *Cassiopea* jellyfish which lack a centralized nervous system (Nath et al., 2017). Sleep and wake have opposing effect on brain activity as wake leads to a net synaptic potentiation (Vyazovskiy et al., 2017), while global synaptic depression occurs during sleep (Vyazovskiy et al., 2008). The core claim of SHY hypothesis is that the function of sleep is the restoration of synaptic homeostasis due to the synaptic downscaling that resets the strengthening of the synapses occurring during wake (Tononi and Cirelli, 2003, 2012, 2019).

Sleep is regulated by two processes: a homeostatic process (S) reflecting the accumulated need for sleep during wakefulness interacting with a process (C) controlled by the circadian pacemaker (Borbely et al., 2016). The interaction between process S and process C determines the timing, duration and quality of sleep (Dijk and Franken, 2005). Sleep homeostasis is strongly correlated with slow wave activity (SWA) with frequencies ranging between 0.5 and 4.5 Hz during NREM sleep (Deboer, 2007). Sleep homeostasis is responsible for the compensatory rebound sleep following sleep deprivation (SD), resulting in increased duration and/or deepening of subsequent sleep via elevated SWA power during NREM sleep (Sanford et al., 2015; Allada et al., 2017). NREM sleep is dominated by slow waves (SWs) when cortical pyramidal cells and interneurons alternate between sustained firing during the up state and hyperpolarization, characterized by neuronal silencing, during the down state. The transitions between the up and down states are associated with cortical slow waves and prolonged duration of the down state leads to greater SWA power (Steriade et al., 2001; Battaglia et al., 2004; Vyazovskiy and Faraguna, 2015; Levenstein et al., 2019). Moreover, it is well established that slow waves (SWs) mediate the synaptic downscaling possibly associated with the renormalization of synapses during sleep (Levenstein et al., 2017; Norimoto et al., 2018; Tononi and Cirelli, 2019).

The increase in SWA power during NREM sleep is proportional to the duration of previous waking (Tobler et al., 1992; Huber et al., 2000a). However, the increase in the power of SWA is influenced by factors independent of the waking duration (Deboer, 2015). For instance, in humans, older age is associated with lower sleep efficiency manifested as a more blunted decrease in SWA, or lower decay of process S, during sleep. Moreover, the quality of the waking experience affects the magnitude of SWA following sleep deprivation. For example, a stressful social encounter leads to greater increase in SWA in mice (Meerlo et al., 1997). Additionally, time of the day, lighting conditions and sleep pressure also markedly affect the magnitude of increase in SWA following SD (Tobler et al., 1994; Werth et al., 1996; Deboer et al., 2007; Vyazovskiy et al., 2007). Furthermore, local features influence the magnitude of SWA as homeostatic sleep regulation is not only a global process, but has a clear local aspect due to varying underlying cortical dynamics (Huber et al., 2000b; Borbely, 2009; Zavada et al., 2009; Guillaumin et al., 2018). Indeed, the increase in SWA after wake and its local regulation are some of the corollaries proposed by SHY (Tononi and Cirelli, 2012).

To date, as far as we are aware, only a couple of studies investigated the effect of chronic stress on homeostatic sleep regulation in the context of SWA power during baseline sleep (Olini et al., 2017; Radwan et al., 2021) and after a sleep deprivation challenge (Olini et al., 2017; Radwan et al., 2021). The study reported deficiency in homeostatic SWA rebound response, following SD in the light, in mice exposed to 10-d chronic social defeat (CSD) stress paradigm. No deficiency in baseline SWA was reported. We investigated the regulation of baseline homeostatic sleep by measuring SWA in C57BL/6J mice following 15-d exposure to social stress. Specifically, we studied the differences in sleep homeostasis in baseline sleep and following SD in the dark period, in mice that adapted to stress and were resilient versus mice that did not adapt to stress and were susceptible. SD paradigm was chosen to be performed in the dark as we previously performed the SD paradigm in the light on mice exposed to CSD (Radwan et al., 2021), and we were interested in investigating the difference in the homeostatic response between the different phenotypes given this new context of a different sleep-wake history. Furthermore, topographical variation of homeostatic sleep process was assessed by comparing EEG dynamics recorded from both frontal and parietal areas.

## Materials and Methods

### Ethics Statement

All experiments were approved by the NYUAD Animal Care and Use Committee, and all experimental protocols were conducted according to the National Institute of Health Guide for Care and Use of Laboratory Animals (IACUC Protocol: 150005A2).

### Animals

C57BL/6J male mice (10–16 weeks; Jackson Laboratories, ME, United States) and CD1 male retired breeders (Charles River, United Kingdom) were used in this study. Only male mice are used in this study as we used the conventional version of the CSD paradigm designed for male mice (Golden et al., 2011). Prior to any experiments, all animals were group housed with their respective strains under standard temperature and humidity-controlled conditions (21 ± 2°C and 50 ± 10%, respectively) with access to food and water *ad libitum*, and were maintained on a 12/12-h light-dark (L/D) cycle, lights on at 7:00 AM and lights off at 7:00 PM, zeitgeber time (ZT 0 = lights on, ZT 12 = lights off). Zeitgeber time is a unit of time based on 12:12 light: dark cycle. All behavioral tests were conducted during the light cycle, between ZT04 and ZT09. Additionally, all C57BL/6J used in this study started around the same age (10 ± 2 weeks).

### Chronic Implant Surgery

**FIGURE 1:**
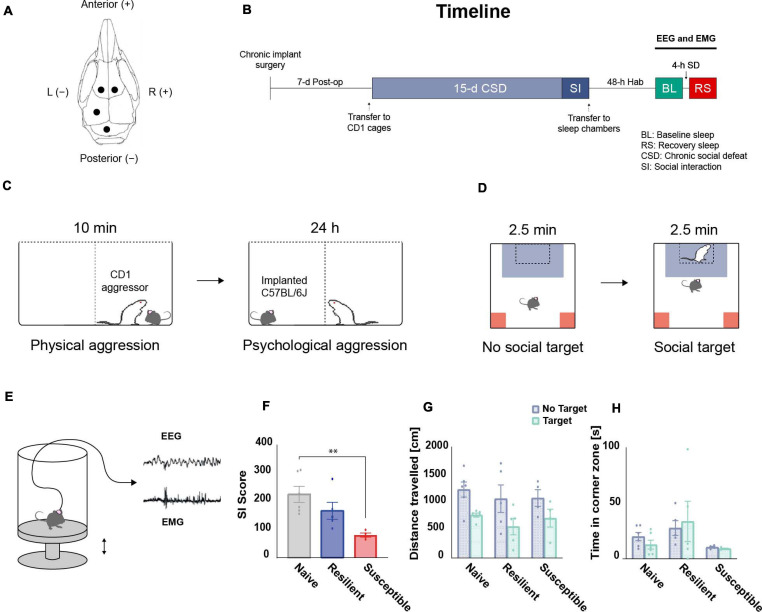
Overview of the experimental design. **(A)** Chronic implantation of EEG electrodes: schematic illustration of the frontal and parietal EEG electrodes arrangement on the skull. **(B)** Timeline: mice were single-housed during the 7-d postoperative recovery period prior to CSD. After 15-d of CSD and a social interaction (SI) test, EEG and EMG baseline (BL) recordings were performed for 24-h after 48-h of habituation (Hab) in the sleep chambers. Post-CSD sleep homeostatic response, following 4-h of sleep deprivation (SD) starting at ZT14, was also acquired. **(C)** CSD paradigm: For 15 days, C57BL/6J mice were exposed to a daily 10-min physical aggression session with a novel retired CD1 breeder. Between sessions, mice were separated by a clear perforated plexiglass divider for the following 24-h allowing for psychological aggression. **(D)** SI test: CSD-exposed mice were classified into stress-resilient and susceptible phenotypes based on their SI score. **(E)** Tethered EEG and EMG recording: Mice were habituated to sleep chambers and connected to an EEG/EMG acquisition system that allowed for free movement in all three dimensions. Sleep deprivation (SD) was executed by sending electric pulses to a magnet placed under the platform which pushes it up and wakes up the mice. **(F)** There was a significant effect of ‘phenotype’ among SI scores using one-way ANOVA (F_2_,_12_ = 6.92, *p* = 0.01). The SI scores of the susceptible mice are lower than the scores of the stress-naïve mice (*p* < 0.01). There were no significant differences in **(G)** total travel distance or **(H)** time in corner zones between phenotypes during the SI test. *n* = 4–6. ^∗^*P* < 0.05, ^∗∗^*P* < 0.01.

Prior to the start of the paradigm, all C57BL/6J mice were implanted for electroencephalogram (EEG) and electromyogram (EMG) recording after reaching 6–8 weeks old age. Mice were anesthetized with an intraperitoneal injection of ketamine-xylazine solution (K: 100 mg kg^–1^, X: 10 mg kg^–1^) and fixed to a stereotactic frame (Kopf Instruments). Their heads were shaved, their scalps opened medially, and the periostea removed. A dental precision driller (Stoelting) was used to drill four holes into the skull, each of which was fit with an electrode for EEG recording in: the left and right frontal lobes (anterior–posterior: + 1.0 mm, medial–lateral: ± 1 mm), the left parietal lobe (anterior–posterior: –2.0 mm, lateral–medial: –1.5 mm), and the cerebellum for grounding/reference (interparietal bone). All coordinates are defined relative to Bregma (Figure 1A). Two additional electrodes were lowered bilaterally into the neck muscle for EMG recording (directly caudal to the occipital bone). All EEG electrodes consisted of stainless-steel screws (Bilaney) with: 2.5-mm head diameters, 1.57-mm shaft diameters, and 1.6-mm shaft lengths. All electrodes were secured to the skull using acrylic C&B Metabond (Parkell Inc.), after which all wires were connected to a female headstage connector (MS 363 Pedestal PlasticsOne). Next, dental cement (Stoelting) was applied around the head connected to protect all the wires and the connector. Following surgery, all mice were allowed to recover single-housed for 7-d postoperative period (Figure 1B).

### Chronic Social Defeat (CSD) Stress Paradigm and Social Interaction (SI) Test

CSD and SI were performed according to previously published protocols (Krishnan and Nestler, 2008; Golden et al., 2011; Chaudhury et al., 2013). Upon arrival, CD1 mice were screened for aggressive behavior and only the aggressive ones were included in the CSD. Aggressive CD1 mice were single-housed in one half of a cage divided in two by a clear, perforated plexiglass partitions, and allowed to habituate for at least 72 h. For a total of 15 days, experimental C57BL/6J mice were introduced daily into the cage of a novel CD1 aggressor for 10 min, during which time C57BL/6J mice were physically attacked by the CD1 aggressors. Any excessive wounding to C57BL/6J due to the physical aggression resulted in the removal of the mouse from the study and immediate euthanasia. Such an incident never occurred in our current study. Moreover, the duration of individual defeat sessions was reduced (from 10 min to 8 min) if the aggression of CD1 was excessive on a given day. Such an incident occurred rarely in our current study. After 10 min of physical aggression, the C57BL/6J mice were separated by a clear perforated plexiglass divider to experience psychological and sensory aggression for 24 h. The health and well-being of the C57BL/6J mice were monitored throughout the CSD. Control C57BL/6J mice were similarly housed in pairs, each in one half of a cage equally divided in two by a clear, perforated plexiglass partition. On the last day of CSD, experimental and control C57BL/6J mice were single-housed in new hamster cages after the last session of physical aggression (Figure 1C).

On recovery day 16, social avoidance behavior toward a novel, non-aggressive CD1 mouse was assessed in a two-trial SI test. In the first, 2.5-min trial, each C57BL/6J mouse was allowed to freely explore a square-shaped arena arena (44 cm × 44 cm) containing an empty, perforated plexiglass cage (10 cm × 6 cm) centered against a wall of the arena (the “No Target” condition). In the second 2.5-min trial, the C57BL/6J mouse was reintroduced into the arena with a novel and non-aggressive CD1 mouse placed in the plexiglass cage (the “Target” condition). The setup was cleaned before and after use and in between trials using MB-10 solution (Quip Laboratories, Inc., United States) to avoid intertrial persistence of olfactory cues. The TopScan video tracking system (CleverSys, Inc.) was used to automatically monitor, compute, and record the amount of time spent in the “interaction zone” (14 cm × 26 cm area around the plexiglass cage) and “corner zone” (10 cm × 10 cm corner areas opposite the cage) and the total distance traveled by each C57BL/6J mouse during the test. These three parameters were collected and analyzed. Classification of experimental C57BL/6J mice into resilient and susceptible subgroups was based on the SI ratio: 100 × Time spent in interaction zone during “Target” condition/Time spent in interaction zone during “No Target” condition. Experimental C57BL/6J mice with SI ratios > (100 + threshold) were classified as resilient; those with SI ratios < (100 − threshold) were classified as susceptible. Threshold was used to avoid using mice with a score close to 100. We set a threshold of 1.0% (Figure 1D).

### Electroencephalogram (EEG)/Electromyogram (EMG) Recording

#### Preprocessing and Visualization

After the post-operative period, all C57BL/6J mice were each transferred to a quasi-soundproof isolation sleep chamber (ViewPoint) also held under the standard laboratory conditions outlined before (21 ± 2°C; 12/12-h L/D cycle, lights on at 07:00). Mice were allowed to habituate 48-h prior to baseline EEG and EMG recording. All mice were connected to a cable that was attached to a rotating commutator (SL-89-Opt-6, Dragonfly, Ridgeley, United States) to allow free movement in all three dimensions during the EEG and EMG recording sessions. Unipolar EEG and bipolar EMG signals were amplified 800× (TBSI, part of HBIO, Cambridge, United States). Digitization was performed using a DAQ card (TBSI, part of HBIO, Cambridge, United States). Data were sampled at 30 kHz and low-pass filtered at 7 kHz, and subsampled at 250 Hz online. The electrophysiological signals were all imported into a custom software program (SleepScore, ViewPoint, Lyon, France) for visualization, scoring, and analysis.

### Electrophysiology Data Analysis

The vigilance states Wake, NREM sleep and REM sleep were visually scored off-line using the EEG and EMG signals according to standard criteria and methods (Franken et al., 1991) with a 5-s scoring window using custom software (SleepScore, Viewpoint, Lyon France). We used both the frontal and parietal EEG signals. The frontal EEG signal was recorded using two bilateral electrodes and we used the signal with the lower signal to noise ratio (SNR) for the analysis. For the NREM states, there is a predominance of oscillations with frequencies ranging from 1 to 5 Hz, that exhibited greater amplitude in the frontal electrode. For the REM states, there is a predominance of oscillations with values ranging from 7 to 10 Hz in the parietal electrode along with simultaneous muscle atonia observed using the differential EMG signal. The wake state is characterized by desynchronization of the oscillations on both the frontal and parietal electrodes, and greater amplitude of the differential EMG signal. Sleep and wake states were visually analyzed and scored by AYT. The analysis was performed blind to eliminate experimenter’s bias. The occurrence of artifacts was very low (∼3–5%) in all of the mice used in the study and concentrated primarily in the wake states (motion-related artifacts). Sleep epochs containing artifacts were excluded from the spectral analysis.

The percent time, average bout duration and the number of bouts were extracted to quantitatively assess the vigilance states. The spectral parameters of the EEG recordings [e.g., power spectral density (PSD)] were computed using the mean spectrum analysis with rectangular windows with 20% overlap of the custom software (SleepScore, Viewpoint, Lyon France). SWA was defined as the EEG power in NREM epochs between 0.5 and 4.5 Hz and was normalized with respect to the median SWA value overall all NREM epochs in the baseline recording (Guillaumin et al., 2018). Further analysis was then performed using custom scripts in Python.

### Sleep Deprivation

After baseline recording, the mice underwent 4-h sleep deprivation in the dark period. The 4-h sleep deprivation was applied on the C57BL/6J mice using the ViewPoint platform system. Sleep deprivation started at 21:00 (ZT14) and ended at 01:00 (ZT18). In this study, we used a basic sleep deprivation paradigm consisting of sending random electric pulses in a randomized sequence to the magnet placed under the platform of the sleep chamber, which pushes it up and wake up the mice. The duration of the pulses was 1,000 ms. The number of pulses per sequence was randomly selected from between three, four, and five pulses. The sequences of pulses were separated by a randomized duration from between 0.15 and 0.27 min (Figure 1E). The use of the current equipment for sleep deprivation offers many advantages: (a) elimination of the human intervention, (b) a standardized random technique to wake up the mice, and (c) the ability to run sleep deprivation on many animals at once (equal to the number of sleep chambers).

EEG and EMG were recorded prior to [i.e., baseline (BL)] and after the deprivation (i.e., recovery): we analyzed the 24-h dark-light (D/L) cycle prior to the deprivation (from 19:00 to 19:00) and the 18-h (D/L) of the cycle post the deprivation (from 01:00 to 19:00).

### Statistical Analysis

To compare the sleep wake profile between the three phenotypes, we used mixed-effects model, instead of two-way repeated measures ANOVA, to account for the missing values (GraphPad Prism, CA, United States). The initial range of normalized SWA (0.5–4.5 Hz) of NREM bouts, corresponding to delta wave, following 4-h SD, was quantified and averaged across 2-h time intervals. Time series of percent time, sleep pressure and SWA power were created by averaging across 2-h time intervals for a 24-h baseline pre-SD or 18-h recovery response post-SD. Time series of the relative cumulative duration of the three vigilance states (wake, NREM, REM) and of relative cumulative frontal and parietal slow wave energy (SWE) were created by averaging across 1-h time intervals for 18-h recovery sleep. To compare between the different time series such as percent time, relative cumulative duration of the three vigilance states (wake, NREM, REM), relative cumulative frontal and parietal slow wave energy (SWE) a mixed-model two-way ANOVA was performed with between-subjects factor ‘phenotype’ (Susceptible vs. Resilient vs. Stress-naïve) and within-subjects factor ‘time’ (12 × 2h) for BL and (9 × 2h) for sleep recovery recordings. Additionally, a mixed-model two-way ANOVA was performed with between-subjects factor ‘phenotype’ (Susceptible vs. Resilient vs. Stress-naïve) and within-subjects factor ‘time’ (6 × 2h) to investigate the light and the dark cycles separately. For *post hoc* analyses on time series and average data, we used Tukey’s multiple comparisons test (*p* < 0.05).

One-way ANOVA was performed with between-subjects factor ‘phenotype’ for average sleep fragmentation. Sleep fragmentation was quantified based on a previously published method (Karamihalev et al., 2019), by multiplying the number of wake bouts by % of sleep. For instance, a greater number of wake bouts for a given sleep duration means that the sleep was more fragmented compared to lower number of wake bouts for the same sleep duration.

To compute SWA buildup across the light and dark period, SWA values in 2-h intervals were log transformed and a simple linear regression analysis was executed (Borbely, 1982a) on Prism, which yielded slope, 95% confidence intervals, standard errors and *p* values. Since sleep in mice is not consolidated, we used wake:sleep ratio in 2-h intervals as a behavioral correlate for sleep pressure. To compute sleep pressure (wake:sleep ratio) buildup, a simple linear regression analysis was executed on Prism. Comparison of the sleep pressure buildup rate between the three phenotypes was performed by following a previously published method (Cumming, 2009). More specifically, we tested the hypothesis that sleep pressure rate in stress-naïve (70.55% per h) is statistically greater than the sleep pressure rate in susceptible mice (17.83% per h). In the event that the confidence intervals overlapped by less than 50%, the rates would be statistically significantly different from each other (*p* < 0.05).

### Testing for Normality

The normality of SWA power data were originally checked using the Shapiro test and SI scores were shown to be normally distributed data based on previous studies (Krishnan and Nestler, 2008; Golden et al., 2011). The rest of the data were average values of quantities (SWA power, Sleep % Time, Wake % Time, fragmentation, relative cumulative NREM, Wake and REM duration and relative SWE) across 2-h intervals. Therefore, according to the central limit theorem, those average values will be approximately normally distributed.

## Results

### Prolonged Exposure to Chronic Social Stress

The SI score data showed a phenotype effect (F _2_,_12_ = 6.92, *p* = 0.01; Figure 1F) as susceptible mice had lower SI scores than stress-naïve mice (*p* < 0.01, Tukey’s multiple comparisons test; Figure 1F). There were no significant differences in total travel distance in the absence or presence of a target (no target: F_2_,_12_ = 0.268, target: F_2_,_12_ = 0.426, *p* > 0.05 for both; Figure 1G), and in time in corner zones during the SI test (no target: F_2_,_12_ = 2.999; target: F_2_,_12_ = 1.445, *p* > 0.05 for both; Figure 1H).

### Deficient Homeostatic Sleep Regulation in Baseline Sleep in Stress-Exposed Mice Post Long Exposure to Chronic Stress

**FIGURE 2:**
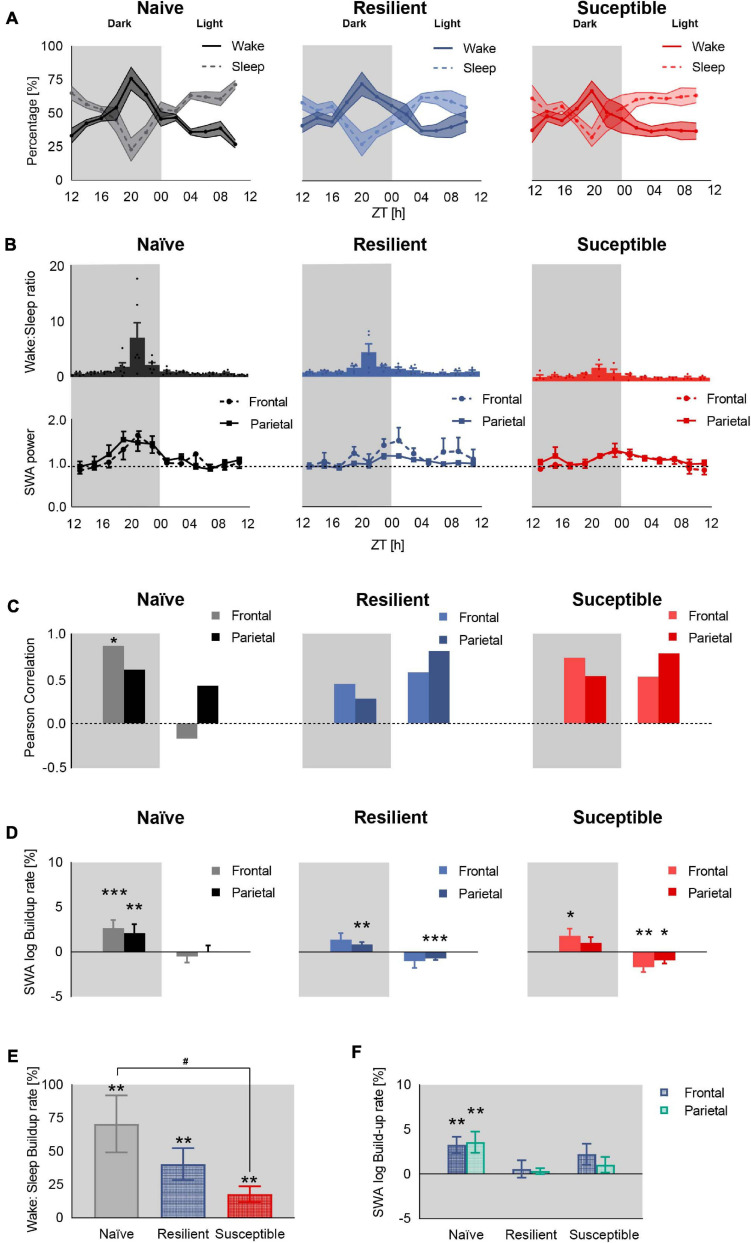
Deficient sleep homeostasis during baseline sleep in stress-exposed mice. **(A)** Percent time in sleep-wake state in stress-naïve, resilient, and susceptible mice. **(B)** Sleep pressure quantified as wake:sleep ratio (top) and frontal and parietal baseline SWA power (0.5–4.5 Hz, bottom). SWA value was normalized to the 24-h baseline median value of SWA. In the dark, the sleep pressure across all phenotypes peaked by ZT20, while frontal SWA peaked at ZT20 in stress-naïve only (*p* = 0.006). **(C)** Pearson correlation between mean SWA power and mean sleep pressure (wake:sleep ratio). *Dark*: There was a positive correlation between mean frontal SWA power and mean sleep pressure in the stress-naïve mice (r_6_ = 0.87, *p* = 0.026). *Light:* There was a trend of significant positive correlation between mean parietal SWA power and mean wake:sleep ratio in resilient and susceptible mice (r_6_ = 0.81, *p* = 0.051 and r_6_ = 0.79, *p* = 0.064 respectively). **(D)** Baseline SWA build-up rate. Each bar represents the coefficient of regression between log SWA (dependent variable) and time (independent variable) in the dark and light separately. *Dark:*There was a significant increase in the buildup of frontal and parietal SWA in stress-naïve mice in the dark (*p* < 0.001 and *p* = 0.002). The buildup of parietal SWA was significant in resilient (*p* = 0.006) and the buildup of frontal SWA was significant in susceptible mice (*p* = 0.04). *Light:* The parietal SWA power decayed in resilient mice (*p* < 0.001) while both frontal and parietal SWA power decayed in susceptible mice (*p* = 0.006 and *p* = 0.01 respectively). **(E)** The buildup rate of sleep pressure (wake:sleep ratio) from the beginning of the dark period (ZT12) to maximum wake:sleep ratio, reversal point at ZT20, was significant across all phenotypes (*stress-naïve*: *p* = 0.003, *resilient*: *p* = 0.003, *susceptible*: *p* = 0.007). The buildup rate of stress-naïve is significantly higher than the buildup rate of susceptible mice (*p* < 0.05). **(F)** Only stress-naïve mice displayed a significant buildup rate of frontal and parietal SWA within ZT12 to ZT20 (*p* = 0.001 and *p* = 0.005). Values are expressed as mean ± sem across 2-h intervals **(A,B)**. *n* = 4–6 for each group. ^*,#^*P* < 0.05, ^∗∗^*P* < 0.01, and ^∗∗∗^*P* < 0.001.

In order to assess the homeostatic sleep process (Process S) across the three phenotypes, we attempted to define a ‘behavioral’ correlate of sleep pressure, which increases during wake and decreases during sleep (Borbely et al., 2016). We first computed the percent time spent in wake and sleep (NREM + REM) in all three phenotypes post-CSD across 2-h intervals (Figure 2A and Supplementary Figure 1). Since sleep in rodents is not consolidated, we quantitatively assessed sleep pressure by computing the ratio between percent time of wake to percent time of sleep (wake:sleep ratio) across the 2-h intervals and used this ratio as a behavioral correlate (Figure 2B top). The wake:sleep ratio peaked at ZT20 corresponding to the point of reversal of the percent time spent in wake and sleep (Figure 2A). Similarly, frontal and parietal SWA in stress-naïve mice gradually increased during the early dark cycle and peaked around ZT20 (*p* = 0.001 and *p* = 0.005), after which they started declining toward the end of the dark cycle and remained low during the light cycle when the sleep pressure is low due to greater amount of sleep relative to wake (Figure 2B bottom). In resilient mice, the frontal SWA peaked at the end of the dark cycle (*p* = 0.06), which is delayed relative to the peak of wake:sleep ratio. In contrast, parietal SWA in resilient mice was more blunted, but also increased toward the end of the dark cycle (*p* = 0.006). In susceptible mice, frontal and parietal SWA were more blunted, but frontal SWA power increased by the end of the dark cycle (*p* = 0.04). Next, we quantified the temporal correlation between mean wake:sleep ratio (Figure 2B top) and mean SWA power (Figure 2B bottom). Since SWA of NREM sleep is a proxy for sleep homeostasis and a functional correlate of sleep pressure, we hypothesized that the increase in SWA power from early- to late-dark period should be consistent with the increase in sleep pressure (wake:sleep ratio), during the regular waking hours of mice in the dark. Thus, we computed the correlation between wake:sleep ratio and SWA power in the dark and light periods separately (Figure 2C). There was a strong positive correlation between sleep pressure and frontal SWA power in the dark in stress naïve (*p* < 0.05), but not in resilient and susceptible, mice. Conversely, there was no correlation between parietal SWA and wake:sleep ratio in all phenotypes in the dark. In the light, the wake:sleep ratio was low for all phenotypes. Only resilient and susceptible mice displayed a trend showing positive correlation between sleep pressure and parietal SWA power in the light (*p* = 0.051 and *p* = 0.064 respectively). Next, in order to quantitatively assess the build-up of SWA in the light and dark periods separately, SWA was log-transformed (Borbely and Wirz-Justice, 1982) and a simple, least-squares linear regression was fit to the dark and light period separately(Figure 2D). *In the dark*, there was a significant buildup of frontal SWA in stress-naïve and susceptible mice (*p* < 0.001 and *p* = 0.04 respectively). There was a trend of increased frontal SWA power in resilient mice (*p* = 0.06). There was a significant increase in parietal SWA power in stress-naïve and resilient mice (*p* = 0.002 and *p* = 0.006). *In the light*, there was a significant decay in frontal SWA power in susceptible mice (*p* = 0.006). Moreover, parietal SWA decayed significantly in resilient and susceptible mice (*p* < 0.001 and *p* = 0.01). In stress-naïve mice, the decay in frontal and parietal SWA power started towards the end of the dark cycle at ZT20, (*p* = 0.0015 and *p* = 0.004 respectively) and remained low during the light cycle with no change.

Our results demonstrate that the buildup of SWA power occurs in the dark- and the light-period is characterized with low or decaying SWA power. We next used a simple way to further explore the concordance between the buildup in sleep pressure and SWA power in the dark since the correlation analysis performed above involved using a window over the whole dark and light period. We were interested in demonstrating that a significant buildup of wake:sleep ratio from the beginning of the dark period (ZT12) to ZT20, when a reversal in the sleep and wake profile occurred across all phenotypes (increased sleep pressure), was associated with a significant buildup of SWA power specifically within the same time interval in the dark. Therefore, we computed the buildup rate, as described above by extracting the slope from the simple linear regression, of sleep pressure (Figure 2E) and SWA power (Figure 2F) from the beginning of the dark period (ZT12) to ZT20. The three groups of mice displayed a buildup of sleep pressure from the beginning of the dark period up to ZT20 (*stress-naïve*: *p* = 0.003, *resilient*: *p* = 0.003, *susceptible*: *p* = 0.007) (Figure 2E). The buildup rate of sleep pressure is greater in stress-naïve compared to susceptible mice (*p* < 0.05) since their confidence level overlapped by less than 50% (Cumming, 2009) (Stress-naïve 95% CI: 0.2681–1.143, Susceptible 95% CI: 0.0546–0.3019). However, the buildup rate of both frontal and parietal SWA during the same time interval (ZT12 to ZT20) was only significant in stress-naïve mice (*p* = 0.001 and *p* = 0.005). In conclusion, both resilient and susceptible mice possess the machinery to buildup SWA power during the dark, similar to stress-naïve mice as demonstrated by the significant buildup of SWA power in the dark across all phenotypes. However, the buildup of SWA was not temporally correlated with the buildup of sleep need as there was a lack of significant buildup of SWA power in resilient and susceptible mice when the sleep pressure was at its peak (ZT20).

### Frontal SWA Power Is Negatively Correlated With Sleep Fragmentation in the Dark Period

**FIGURE 3:**
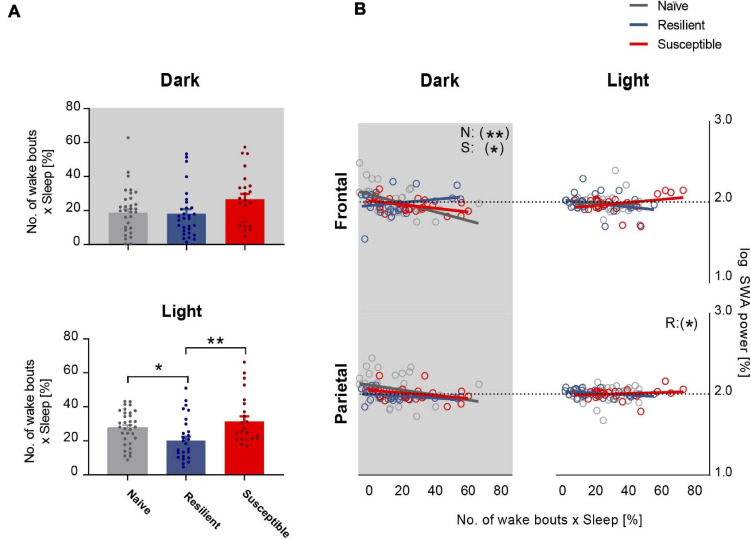
Frontal SWA power is negatively correlated with sleep fragmentation. **(A)** Quantification of sleep fragmentation based on previously published method (Karamihalev et al., 2019) displayed a trend of a phenotypic effect in the dark and an effect in the light (*p* = 0.0534 and *p* < 0.01 respectively). *Dark:* Trend showing greater fragmentation in susceptible mice versus resilient and stress-naïve mice (*p* = 0.07 and *p* = 0.08 respectively). *Light:* Sleep fragmentation in resilient mice was lower than in susceptible and stress-naïve mice (*p* < 0.01 and *p* < 0.05 respectively). **(B)** Correlation between SWA power and sleep fragmentation: There was a significant negative association between sleep fragmentation and frontal SWA in stress-naïve and susceptible mice in the dark (Stress-naive: r_36_ = -0.51, *p* = 0.002; Susceptible: r_23_ = -0.50, *p* = 0.02). Resilient mice exhibited significant negative association between sleep fragmentation and parietal SWA (r_30_ = -0.38, *p* = 0.04) in the light. Values are expressed as mean ± sem across 2-h intervals of sleep fragmentation and log SWA power in the correlation analyses. *n* = 4–6 for each group. ^∗^*P* < 0.05, ^∗∗^*P* < 0.01.

We next investigated the relationship between sleep quality and the power of SWA. We computed the sleep fragmentation in 2-h intervals based on a previously published method (Karamihalev et al., 2019). In short, sleep fragmentation is expressed as the number of wake bouts corrected by the sleep amount. In the dark, there was a trend toward significant difference in sleep fragmentation between the phenotypes (F_2_,_87_ = 3.03, *p* = 0.0534). There was a trend showing greater fragmentation in susceptible mice versus resilient and stress-naïve groups (*p* = 0.07 and *p* = 0.08 respectively, Tukey’s multiple comparisons test; Figure 3A top). In the light, there was a strong difference in sleep fragmentation between the phenotypes (F_2_,_85_ = 5.88, *p* = 0.004). Sleep fragmentation in resilient mice was lower than in susceptible and stress-naïve mice (*p* < 0.01 and *p* < 0.05 respectively, Tukey’s multiple comparisons test; Figure 3A bottom).

Lower frontal SWA power was associated with greater sleep fragmentation in the dark in stress-naïve and susceptible mice (*p* = 0.002 and *p* = 0.02 respectively). Nonetheless, there was no association between frontal SWA power and sleep fragmentation in the light. Additionally, there was also a lack of association between parietal SWA and sleep fragmentation except for the light period in resilient mice when parietal SWA was negatively correlated with sleep fragmentation (*p* = 0.04) (Figure 3B).

### Deficient Sleep Recovery Response in Susceptible Mice Post 4-h SD in the Dark

**FIGURE 4:**
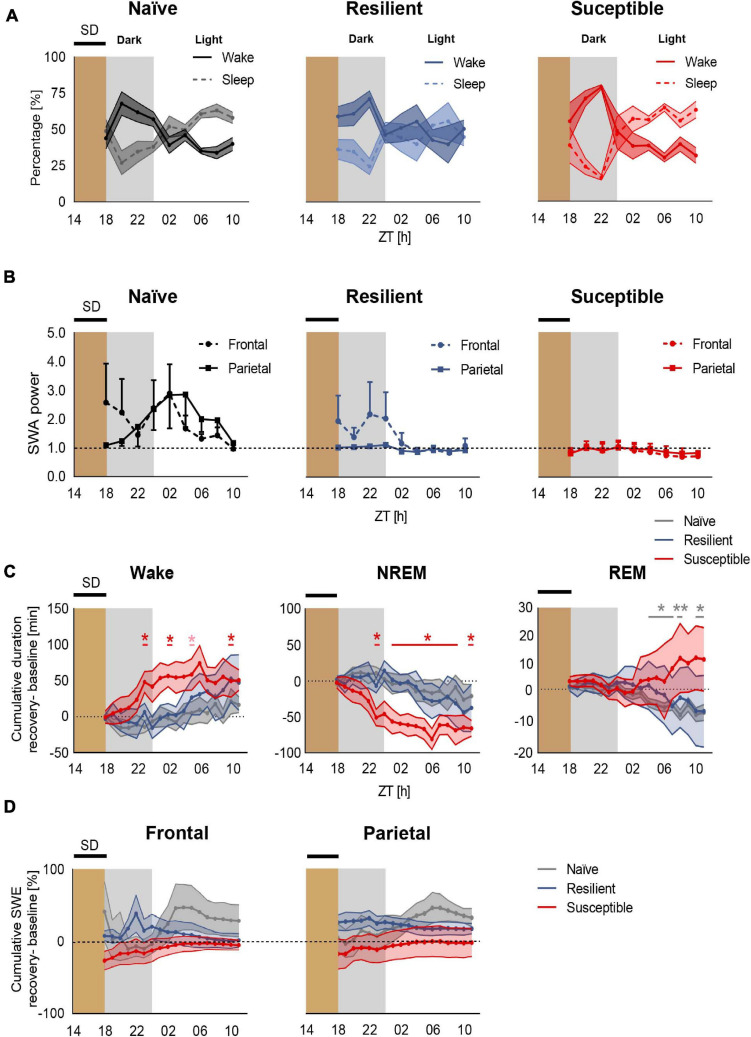
Deficient recovery sleep response post SD applied in the dark in susceptible mice. **(A)** Recovery sleep-wake response*:* Percent time of sleep and wake in stress-naïve, resilient, and susceptible mice post SD. **(B)** Homeostatic SWA (0.5–4.5 Hz): Frontal and parietal SWA power of stress-naïve, resilient, and susceptible mice. Homeostatic SWA value was normalized to the 24-h pre-SD baseline median value of SWA. 4-h SD elicited mild non-significant increase in frontal SWA power in stress-naïve and resilient mice. Frontal and parietal SWA power were blunted and less variable in susceptible mice. **(C)** Cumulative difference in duration of vigilance states was calculated by subtracting the cumulative *baseline* duration from the cumulative *recovery* duration. *Left:* Change in cumulative duration of wake post SD. Susceptible mice spent a significantly greater amount of time in wake (one-sample t tests; ZT23: *p* = 0.044, ZT02 *p* = 0.030, ZT05: *p* = 0.057, ZT10: *p* = 0.038). *Middle:* Change in cumulative duration of NREM sleep post SD. Susceptible mice lost a significant amount of NREM sleep (one-sample t tests; ZT23 and ZT01–ZT10: *p* < 0.05; ZT11: *p* < 0.01). *Right:* No change in cumulative duration of REM post SD in stress-exposed. However, there was a negative change in the cumulative duration of REM in stress-naïve mice post SD (one sample *t*-tests, ZT04–ZT07: 0.021 < *p*-values < 0.0345; ZT08: *p* = 0.0042, ZT10: *p* = 0.0137, ZT11:*p* = 0.0336). **(D)** Percent change in cumulative slow wave energy (SWE) post SD in the frontal (left) and parietal (right) leads. Two-way repeated measures ANOVA in the light and in the dark showed no difference in the intensity of the sleep in either frontal or parietal SWE between the three phenotypes. Values are expressed as mean ± sem across 2-h intervals **(A,B)** and 1-h intervals **(C,D)**. *n* = 4–6 for each group. ^∗^*P* < 0.05, ^∗∗^*P* < 0.01.

We next investigated the effect of prolonged exposure to chronic stress on sleep homeostasis following enforced wakefulness. Mice were sleep deprived for 4-h in the dark between ZT14 to ZT18 (Figure 1B and Supplementary Figure 2). We first computed the percent time of wake and sleep (NREM + REM) in all three phenotypes post SD across 2-h intervals (Figure 4A and Supplementary Figure 3). Though not significant, susceptible mice displayed a trend of heightened wakefulness during the first 4–6 h of rebound sleep during the dark compared to stress-naïve and resilient mice. However, a 4-h SD applied in the dark at ZT14 to ZT18 failed to elicit a significant homeostatic increase in frontal or parietal SWA power in mice across all three phenotypes. There was an elevated frontal SWA in both stress-naïve and resilient mice in the dark period immediately after SD. However, the responses were highly variable and not significant. During the light period, both frontal and parietal SWA were elevated (with high variability) in stress-naïve mice (Supplementary Figure 4). In susceptible mice, the homeostatic SWA response was blunted in both the dark and light period (Figure 4B). Despite the lack of significant increase in SWA power, we continued to investigate the homeostatic mechanisms by assessing the changes in the amount of sleep and SWA power which reflects the intensity of NREM sleep (Deboer, 2018). We computed the change in the cumulative duration of NREM sleep during recovery sleep, in 1-h mean values, by subtracting the cumulative duration of NREM sleep in the baseline from the cumulative duration of NREM sleep post SD during the same period. Following SD, susceptible mice spent significantly more time in Wake (Figure 4C, left) and less time in NREM sleep as demonstrated by the positive relative cumulative duration of Wake (one-sample *t* tests; ZT23: *p* = 0.044, ZT02 *p* = 0.030, ZT05: *p* = 0.057, ZT10: *p* = 0.038; Figure 4C, left) and the negative relative cumulative duration of NREM (one sample t-tests, ZT23 and ZT01–ZT10: 0.011 < *p*-values < 0.043; ZT11: *p* = 0.0091; Figure 4C, middle). Repeated measures two-way ANOVA of relative cumulative wake duration post SD yielded significant phenotype × time interaction in the dark (F_10_,_55_ = 2.72, *p* = 0.009, Figure 4C, left). Repeated measures two-way ANOVA of relative cumulative NREM duration post SD yielded significant phenotype x time interaction (F_10_,_60_, *p* < 0.001) and significant time effect in the dark (F_2__.__307_,_27__.__69_, *p* = 0.04; Figure 4C, middle). We next assessed the relative cumulative duration of REM and found no change in REM sleep for stress-exposed mice (Figure 4C, right). However, there was a negative relative cumulative duration of REM in stress-naïve mice post SD (one sample *t*-tests, ZT04–ZT07: 0.021 < *p*-values < 0.0345; ZT08: *p* = 0.0042, ZT10: *p* = 0.0137, ZT11: *p* = 0.0336; Figure 4C, right).

We then assessed the change in SWA power during recovery sleep by computing slow wave energy (SWE) defined as cumulative SWA power, in 1-h mean values, and subtracting the cumulative SWA power in the baseline from the cumulative SWA post SD during the same period. Stress-naïve mice exhibited a mild, non-significant, increase in the frontal SWA power in the dark relative to resilient and susceptible mice. No significant change was detected in frontal SWE between the phenotypes (Figure 4D, left). Moreover, all three phenotypes did not show change in parietal SWE (Figure 4D, right). Repeated measure ANOVA analysis did not yield phenotypic differences for either frontal or parietal relative SWE. In summary, a mild 4-h SD protocol elicited an impaired sleep recovery response as demonstrated by the loss of NREM sleep post SD in susceptible mice.

## Discussion

Here, we show that long exposure to chronic stress is associated with deficient homeostatic sleep process during baseline sleep and following 4-h of mild enforced wakefulness applied during the dark period.

Increasing work demands and social activities in the modern 24/7 society are closely related to sleep derangements. Sleep reactivity refers to the degree that sleep is disrupted following exposure to a given amount of stress (Drake et al., 2014). The differences in sleep reactivity following stress exposure between individuals suggests sleep undergoes complex regulatory control. Multiple factors including the adaptation to stress and chronicity of stressor modulate the sleep response to stress due to the overlap in the mechanisms regulating sleep and stress response (Adrien, 2002; Pillai et al., 2014; Murphy and Peterson, 2015; Kalmbach et al., 2018a,b).

The propensity for wake and sleep states is homeostatically regulated via process S which is correlated to the power of SWA during NREM sleep. The S-deficiency hypothesis refers to aberrant homeostatic regulation of sleep in depression due to a deficiency in the homeostatic buildup of sleep debt during wakefulness (Borbely, 1982a, 1987; Frey et al., 2012a). Since Process S is a determinant of sleep duration and intensity, an aberrant process S may explain the comorbidity of sleep disturbances with depression (Borbely, 1987).

To our knowledge, two studies investigated the effect of chronic social stress on homeostatic sleep regulation (Olini et al., 2017; Radwan et al., 2021). Chronic social defeat (CSD) is a widely adopted animal model of stress-related disorders (Krishnan et al., 2007; Golden et al., 2011). The two studies differed in the duration of the CSD paradigm where a 10-d and a 15-d CSD paradigm were used by the first and second study respectively. The first study showed a blunted homeostatic response, as measured by SWA power, following 4-h SD applied in the beginning of the light period in stress exposed mice without segregating the susceptible from the resilient mice. In the second study, the aberrant homeostatic response following 4-h SD was more specific to the susceptible mice (Radwan et al., 2021). Investigation of post-stress pre-SD baseline SWA in the first study found no difference between stress-exposed and stress-naïve mice. Thus, the authors suggested that exposure to longer duration of chronic social stress, such as a 15-d protocol, might impact SWA during baseline sleep. Consequently, here, we assessed the effect of a long 15-d exposure to chronic social stress, on process S of baseline sleep, by measuring SWA power as a functional correlate. We were also interested in contrasting process S between susceptible and resilient mice as divergence in their ability to adapt to stress might imply differences in sleep reactivity including processes regulating homeostatic sleep. Additionally, we assessed the topographical variation in SWA by comparing EEG recorded from frontal and parietal areas.

The sleep-wake profile of stress-exposed and stress-naïve mice in this study is generally comparable to our previous reports (Radwan et al., 2021). Sleep, in particular NREM sleep, of susceptible mice is more fragmentated in comparison to resilient and stress-naïve mice due to a similar increase in the number of NREM and wake bouts. However, we formerly reported that the sleep-wake profile of resilient mice was more similar to that of stress-naïve mice in the light, while it was more similar to that of susceptible mice in the dark. However, in our current work the sleep-wake profile of resilient mice, in both light and dark, was more similar to that of the stress-naïve mice.

Since sleep is highly fragmented in C57BL/6J mice, we quantitatively assessed the homeostatic buildup in sleep pressure in 2-h intervals by computing the ratio of amount of wake to sleep. The wake:sleep ratio increases in all three groups in the dark period (active phase) and reaches a maximum at ZT20. However, only stress naïve mice exhibited significant buildup of frontal and parietal SWA in the interval ZT12-20. This was confirmed by the positive correlation between frontal SWA and sleep pressure for stress-naïve mice in the dark, while there was no significant correlation in susceptible and resilient mice. It should be noted that the buildup to maximum wake:sleep ratio (sleep pressure) is greater in stress-naïve mice relative to susceptible mice, which infers that changes in sleep and wake profile is more blunted in the susceptible mice. The buildup of sleep pressure was intermediate in resilient mice. Based on these observations, frontal SWA in stress-naïve mice is a physiological measure of the buildup of sleep debt during the active phase (dark period) and a measure of the dissipation of process S as the decay in both sleep pressure and frontal SWA start at ZT20 (Cajochen et al., 1999). It is important to note that the wake:sleep ratio is not only dependent on process S, but also on process C. In other words, both of the sleep pressure and the circadian clock affect the wake:sleep ratio. Thus, the association between SWA power, the functional correlate of process S, and wake:sleep ratio, as a behavioral correlate of sleep pressure, will differ in the dark and light period. Here, we observed a positive association between wake:sleep ratio and frontal SWA power in the dark and not in the light period, in the stress-naïve mice, which implies that wake:sleep ratio might be used as a behavioral correlate for sleep pressure (process S) only in the dark. It is worth mentioning that both resilient and susceptible mice possess the machinery to build up frontal SWA power during the dark, similar to stress-naïve mice, as was demonstrated by the significant buildup of frontal SWA power throughout the dark period across all phenotypes. However, the buildup of frontal SWA was asynchronous with the buildup of sleep need in the dark period in the stress-exposed mice. Interestingly, in resilient and susceptible mice, the decay in parietal SWA power is correlated with the temporal variations of wake:sleep ratio in the light period. Further investigation is required to assess the differential role of frontal and parietal SWA power in relation to sleep debt of different intensity in the dark and light periods independently. Moreover, our data potentially suggests that the sleep impairments induced by chronic stress exposure could be due to either a deficient process S (Borbely, 1982b; Borbely and Wirz-Justice, 1982; Frey et al., 2012b), or a deficient process C due to circadian dysregulation (Agorastos et al., 2019; Agorastos and Olff, 2020), or a discoordination/asynchrony between both processes.

Our data did not support the hypothesis that compromised sleep efficiency post chronic stress exposure leads to impairment of process S. Sleep fragmentation calculated in 2-h intervals bins was negatively correlated in the dark period with frontal SWA power in stress-naïve and susceptible mice. Indeed, this negative relationship between SWA power and sleep fragmentation is reminiscent of the smaller decay of EEG power during NREM sleep observed in middle-aged humans due to lower sleep efficiency (Dijk and Beersma, 1989). Hence, the impairment of process S could be partially explained by increased sleep fragmentation in susceptible mice. However, such reasoning fails to explain the deficiency of process S in resilient mice, where sleep fragmentation is lower than in susceptible mice, and thus infers that chronic stress-induced impairment of process S is not a consequence of increased sleep fragmentation alone. Additionally, the impairment of process S reported in our study revolved around the synchrony of SWA power with wake:sleep ratio, rather than the amplitude of SWA power *per se*.

We next investigated homeostatic sleep regulation across the phenotypes following a sleep challenge consisting of 4-h SD. Previous studies investigated process S in mice exposed to CSD post SD in the *light* period (Olini et al., 2017; Radwan et al., 2021). Thus, we investigated differences in rebound sleep across phenotypes following exposure to SD in the *dark* period, which is characterized by the predominance of wake. We expected a less pronounced homeostatic response, in terms of how long it would be sustained, compared to the homeostatic response post SD in the light period, when sleep is predominant and the SWA increase lasts for 6–8-h post-SD. Additionally, we had previously shown that the sleep-wake profile of resilient and susceptible mice were similar especially in the second half of the dark cycle post-CSD (Radwan et al., 2021). Therefore, we predicted that the sleep recovery response of resilient mice would be impaired, similar to that of susceptible mice post SD in the dark. In contrast the sleep recovery response, as measured by increased SWA, of the resilient mice post SD in the light was more similar to that of stress-naïve mice. We shifted the 4-h SD window away from the Lights OFF time at ZT12 to ZT14, based on observations that the wake:sleep ratio was still low at this point. All three phenotypes exhibited attenuated rebound sleep, in terms of SWA increase in power, possibly due to the short SD duration (4-h) and the 2-h of sleep that the mice experienced in the beginning of the dark period. This suggests that a longer SD in the dark might induce a robust homeostatic response. Since our 4-h SD paradigm in the dark was ineffective in inducing an increase in SWA power, which is the characteristic of the homeostatic response, this highlights one of the key limitations in our study. Thus, we were unable to test our hypothesis that the impairment of homeostatic rebound in resilient and susceptible mice would be comparable. This is in contrast with the effectiveness of the 4-h SD in the light period, where the SWA increase was greater in stress-naïve mice relative to susceptible even 8-h post SD (Radwan et al., 2021). Interestingly though, susceptible mice, spent less time in NREM and more time in wake post SD in the dark relative to baseline. These findings suggest that mild sleep perturbations applied in the dark, SD, resulted in gradual loss of NREM sleep in susceptible mice, which suggests a deficiency in their homeostatic mechanisms leading them to be more prone to mild external disturbances. Indeed, there is converging evidence that chronic stress exposure impairs synaptic plasticity in the brain and is associated with maladaptive responses to stress and external challenges (Cerqueira et al., 2007; Negrón-Oyarzo et al., 2016; Murphy-Royal et al., 2020). The impaired recovery response post a mild SD challenge exhibited by susceptible mice is in line with those findings, while the sleep of resilient mice was more robust to mild perturbations as they likely undergo homeostatic adaptations that might buffer against some of the stress-induced sleep impairments (Krishnan et al., 2007; Friedman et al., 2014). We would like to point out that our small sample size, particularly for susceptible mice (*n* = 4), added another limitation to our study as it might have made it more challenging to detect additional statistically significant differences in process S and sleep recovery response between the phenotypes.

Mounting evidence indicates that sleep homeostatic regulatory process is a local phenomenon modulated by use-dependent local cortical mechanisms as demonstrated by topographical variation of EEG dynamics of NREM sleep following SD (Huber et al., 2000b; Guillaumin et al., 2018). Difference between frontal and parietal SWA in our data supports the evidence of the existence of topographical variations in EEG signals. Frontal SWA appears to correlate with the wake:sleep ratio only in the dark. In contrast, we did not detect any correlation in parietal SWA in the dark which implies a differential, and currently not well understood, functional role of both signals in process S.

Chronic stress exposure may affect components that regulate sleep homeostasis such as the sensors, integrators or effectors (Cannon and Rosenberg, 1932). The buildup rate of the wake:sleep ratio was higher in stress-naïve mice relative to susceptible mice, which infers a potential impairment in the input signal to the sensor component of process S, and might lead to impairment in the remaining two components of the process downstream of the sensor component. The buildup rate of wake:sleep ratio in resilient mice was intermediate between stress-naïve and susceptible mice. The sensor component of process S is mediated by the release of adenosine in the basal forebrain and controlled by the activity of glutamatergic neurons (Porkka-Heiskanen et al., 1997, 2000; Basheer et al., 2004; Greene et al., 2017; Peng et al., 2020). Future studies would need to investigate the difference in the dynamics of basal forebrain function between stress-exposed and stress-naïve mice.

In summary, exposure to chronic stress leads to asynchrony between the functional correlate of process S (frontal SWA power) and the behavioral correlate of process S in the dark (wake:sleep ratio). Additionally, the pathological behavioral state of susceptibility to stress was further associated with inability to cope with sleep challenges such as acute, mild SD. In conclusion, our findings highlight the need for further studies to explore the finer details of the spatio-temporal dynamics of process S and the variations of such dynamics following prolonged exposure to chronic social stress.

## Data Availability Statement

The raw data supporting the conclusions of this article will be made available by the authors, without undue reservation.

## Ethics Statement

The animal study was reviewed and approved by NYUAD Animal Care and Use Committee.

## Author Contributions

BR and AYT contributed equally and analyzed the data. BR and DC designed the experiments and wrote the manuscript. AYT and SH collected the data. All authors contributed to the article and approved the submitted version.

## Conflict of Interest

The authors declare that the research was conducted in the absence of any commercial or financial relationships that could be construed as a potential conflict of interest.
