# Supplementary material for: Prolonged Exposure to Social Stress Impairs Homeostatic Sleep Regulation
Source: Front Neurosci. 2021 Feb 22;15:633955. doi: 10.3389/fnins.2021.633955 (PMC7937905; doi:10.3389/fnins.2021.633955)
Supplement: Supplementary file 1 [file Presentation_1.pdf]

Supplementary Material

1.1 Supplementary Figures

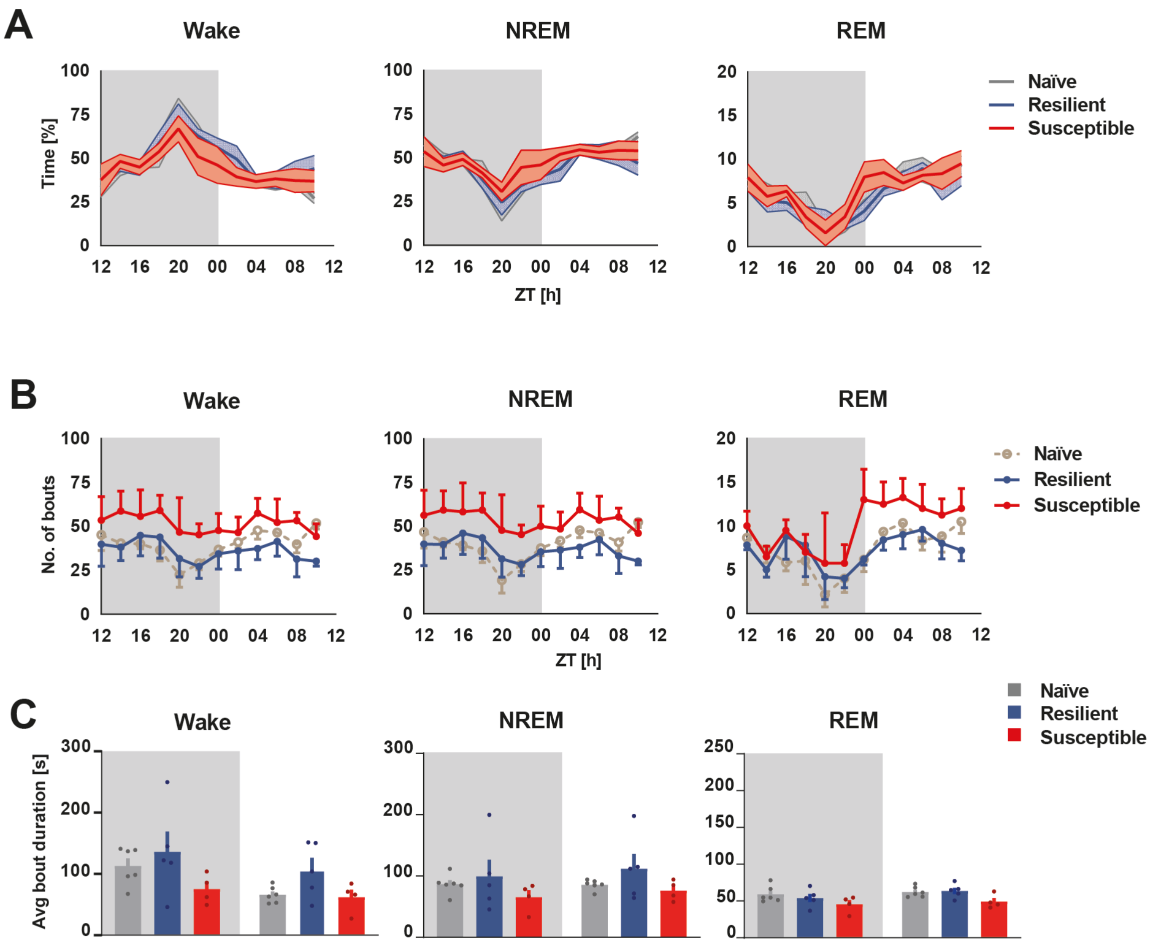

Supplementary Figure 1. Baseline sleep-wake architecture in stress-naïve, resilient and susceptible mice post-CSD. **(A)** Percent of time that stress-naïve, resilient, and susceptible mice spent in Wake, NREM and REM in the dark and light cycle. There was a significant effect of 'time' in all vigilance states ($p < 0.0001$). **(B)** Number of bouts of Wake, NREM and REM post-CSD in stress-naïve, resilient and susceptible mice. There was a significant effect of 'time' in Wake ($F_{5.120,61.44}= 2.76$, $p < 0.05$), in NREM ($F_{5.086,61.04}= 2.95$, $p < 0.05$) and in REM ($F_{3.42, 40.97} = 4.70$, $p < 0.01$). **(C)** Average duration of Wake, NREM and REM bouts post-CSD in all three phenotypes. There was a phenotype effect in Wake ($F_{2,12}=4.25$, $p<0.05$). There was a trend for a phenotype effect in NREM ($F_{2,12}=3.55$, $p=0.06$) and a trend for a 'phase' effect in REM ($F_{1,12}=4.53$, $p=0.055$). Values are expressed as mean ± sem across 2-h intervals **(A - B)** and across the dark and light period separately **(C)**. n= 4-6 for each group.

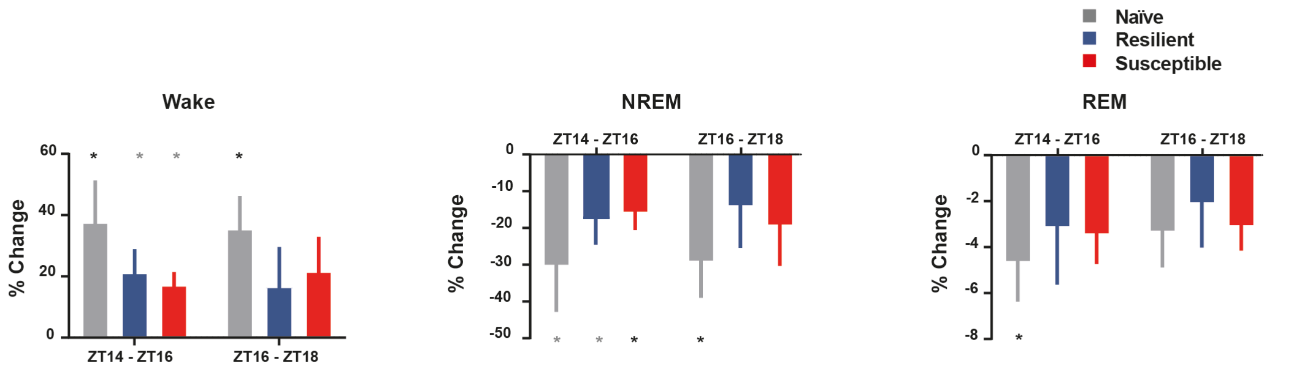

Supplementary Figure 2.

Change in % time of the vigilance states induced during the SD paradigm. Change in % time was computed by subtracting % time of vigilance states in baseline from % time of the corresponding vigilance states during SD. During SD at ZT14-16, there was an increase in % time in wake (Stress-naïve: $p<0.05$, Resilient: $p=0.05$ and Susceptible: $p=0.06$ respectively), accompanied by a decrease in % time in NREM (Stress-naïve: $p=0.058$, Resilient: $p=0.056$ and Susceptible: $p<0.05$ respectively) and a decrease in % time in REM ($p<0.05$) in stress-naïve mice. During SD at ZT16-18, there was an increase in % of time of Wake and a decrease in % time of NREM in stress-naïve mice ($p<0.05$ for both). Values are expressed as mean ± sem across 2-h intervals.

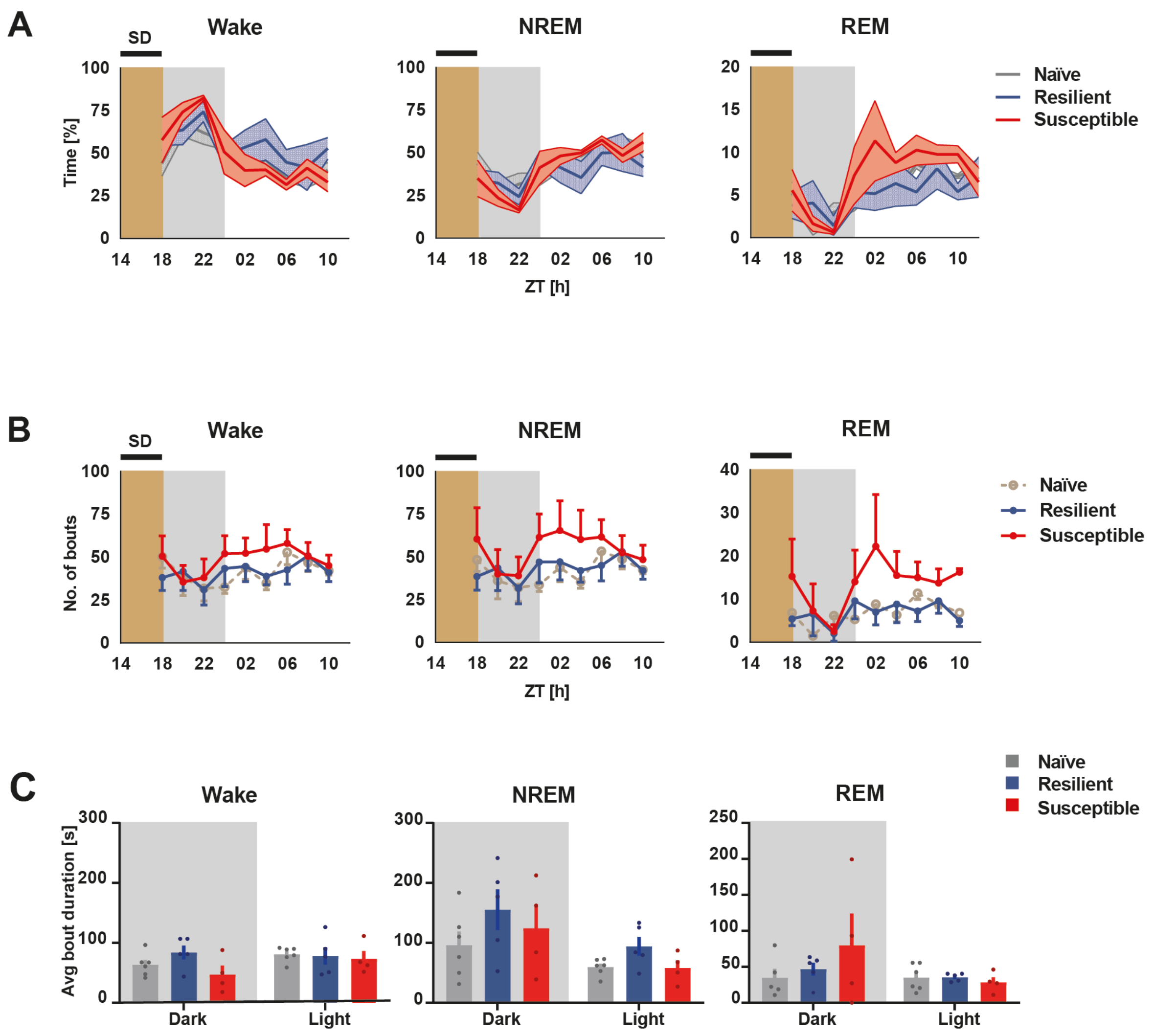

Supplementary Figure 3.

Recovery sleep-wake architecture in stress-naïve, resilient and susceptible mice post-SD. **(A)** Percent of time that stress-naïve, resilient, and susceptible mice spent in Wake, NREM and REM in the dark and light cycle. There was a significant effect of 'time' in all vigilance states ($p < 0.001$). **(B)** Number of bouts of Wake, NREM and REM post-CSD in stress-naïve, resilient and susceptible mice. There was a significant effect of 'time' in all vigilance states ($p < 0.001$). There was a significant interaction between 'phenotype' × 'time' in number of REM bouts in susceptible mice ($F_{2,12} = 4.96$, $p < 0.05$). Additionally, susceptible mice exhibited a significantly greater number of REM bouts compared to stress-naïve (Tukey's multiple comparisons test, $p < 0.05$) and a trend showing greater number of REM bouts compared to resilient mice ($p=0.05$) during the light phase. **(C)** Average duration of Wake, NREM and REM bouts post-CSD in all three phenotypes. There was a trend of phase effect in Wake ($F_{1,12}=4.65$, $p=0.05$). There was a phase effect in NREM ($F_{1,12}=13.86$, $p<0.01$). Values are expressed as mean ± sem across 2-h intervals **(A - B)** and across the dark and light period separately **(C)**. n= 4-6 for each group.

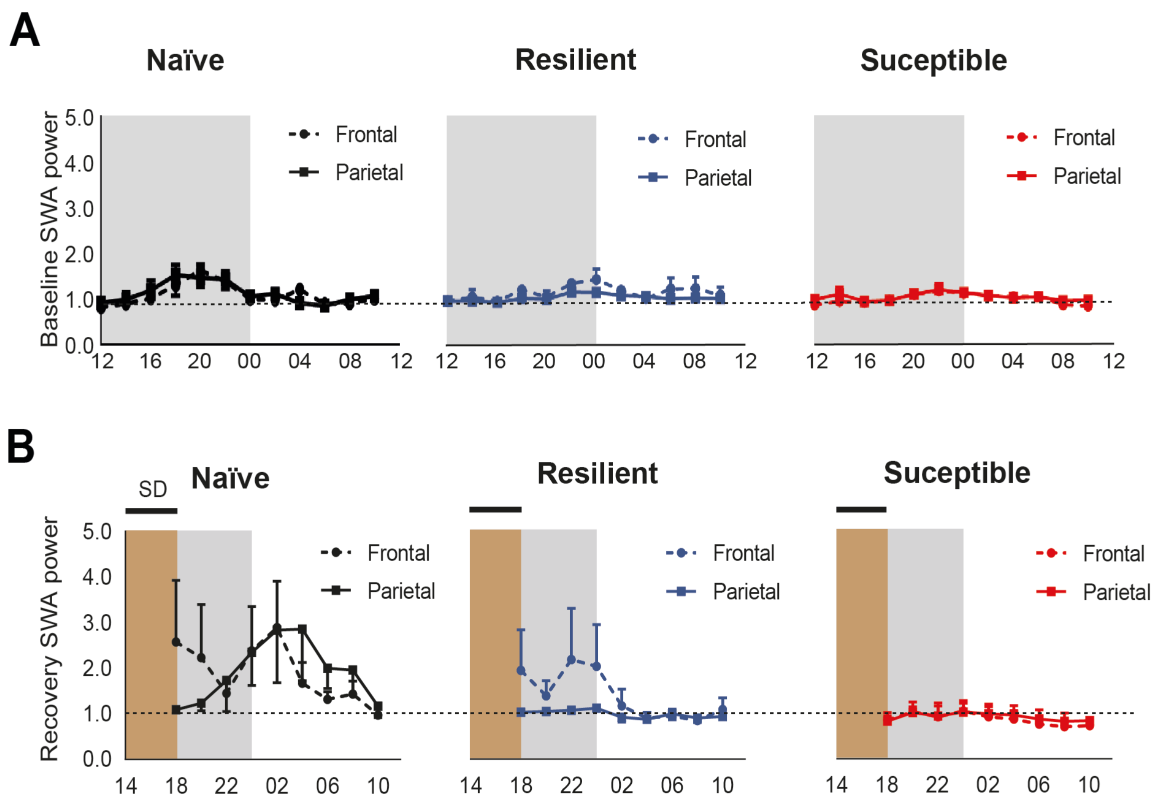

Supplementary Figure 4.

A qualitative comparison between baseline (post-stress pre-SD) **(A)** and recovery (post-stress post-SD) **(B)** SWA power across the three phenotypes by using a similar y-axis scale. The graphs with their corresponding statistics are presented in Figure 2B (bottom) and Figure 4B. For both graphs, SWA value was normalized to the 24-h baseline median value of SWA. Values are expressed as mean ± sem across 2-h intervals **(A - B)**. n= 4-6 for each group.
